# Frailty in people with rheumatoid arthritis: a systematic review of observational studies

**DOI:** 10.12688/wellcomeopenres.17208.1

**Published:** 2021-09-23

**Authors:** Peter Hanlon, Holly Morrison, Fraser Morton, Bhautesh D Jani, Stefan Siebert, Jim Lewsey, David McAllister, Frances S Mair

**Affiliations:** 1 Institute of Health and Wellbeing, University of Glasgow, Glasgow, UK; 2 Institute of Infection, Immunity & Inflammation, University of Glasgow, Glasgow, UK

**Keywords:** Rheumatoid arthritis, frailty, epidemiology

## Abstract

**Background: **Frailty, an age-related decline in physiological reserve, is an increasingly important concept in the management of chronic diseases. The implications of frailty in people with rheumatoid arthritis are not well understood. We undertook a systematic review to assess the prevalence of frailty in people with rheumatoid arthritis, and the relationship between frailty and clinical outcomes.

**Methods: **We searched three electronic databases (January 2001 to April 2021) for observational studies assessing the prevalence of frailty in adults (≥18 years) with rheumatoid arthritis, or analysing the relationship between frailty and clinical outcomes in the context of rheumatoid arthritis. Titles, abstracts and full texts were assessed independently by two reviewers. Study quality was assessed using an adapted Newcastle-Ottawa Scale.

**Results: **We identified 17 analyses, from 14 different sample populations. 15/17 were cross-sectional. These studies used 11 different measures of frailty. Frailty prevalence ranged from 10% (frailty phenotype) to 36% (comprehensive rheumatologic assessment of frailty) in general adult populations with rheumatoid arthritis. In younger populations (<60 or <65 years) prevalence ranged from 2.4% (frailty phenotype) to 19.9% (Kihon checklist) while in older populations (>60 or >65) prevalence ranged from 31.2% (Kihon checklist) to 55% (Geriatric 8 tool). Frailty was associated with higher disease activity (10/10 studies), lower physical function (7/7 studies), longer disease duration (2/5 studies), hospitalization (1/1 study) and osteoporotic fractures (1/1 study).

**Conclusion: **Our review found that frailty is common in adults with rheumatoid arthritis, including those aged <65 years, and is associated with a range of adverse features. However, these is substantial heterogeneity in how frailty is measured in rheumatoid arthritis. We found a lack of longitudinal studies making the impact of frailty on clinical outcomes over time and the extent to which frailty is caused by rheumatoid arthritis unclear.

## Introduction

Rheumatoid arthritis is the most common chronic inflammatory arthropathy, the incidence of which increases with age ^1– 3^. While advances in treatment of rheumatoid arthritis have resulted in marked improvement in outcomes and prognosis, rheumatoid arthritis continues to cause significant symptom burden, loss of function, morbidity, and reduced quality of life ^1, 3^. Frailty has been highlighted as an emerging concept in our understanding of the impact of musculoskeletal disorders ^2^. Frailty is an age-related state of increased vulnerability leading to decompensation in response to physiological stress ^4^. While most studies have focused on people aged over 65 years, frailty is also prevalent and associated with adverse health outcomes in younger populations ^5^. Many measures exist to quantify frailty, of which the most widely used are the frailty phenotype ^6^ (a physical measure assessed by grip strength, walking speed, exhaustion, weight loss, and low physical activity) and the frailty index ^7, 8^ (a cumulative count of age-related deficits including long term conditions, symptoms, functional limitation and physiological markers). Both constructs have potential overlap with features associated with rheumatoid arthritis.

Despite a rapid expansion of frailty research in the last two decades, including in the context of specific index conditions ^9^, research on frailty in the context of inflammatory diseases in general, and rheumatoid arthritis in particular, is relatively recent ^2, 10^. Frailty has been reported to be prevalent in people with rheumatoid arthritis, including relatively young individuals (i.e. <65 years) ^11^. Others have explored associations between frailty and functional limitations in rheumatoid arthritis ^12^. However, the diversity of measures used to quantify frailty, and overlap between features of rheumatoid arthritis and frailty constructs, means that understanding the relationship between frailty and rheumatoid arthritis requires careful consideration. 

This systematic review seeks to synthesise data from observational studies of frailty in people with rheumatoid arthritis. We aim to assess (i) what frailty measures have been used in published studies including people with rheumatoid arthritis, (ii) what is the prevalence of frailty in people with rheumatoid arthritis across a range of ages, (iii) what is the association between frailty and features of rheumatoid arthritis such as disease activity, functional limitation, and duration, and (iv) if frailty is associated with adverse health outcomes in the context of rheumatoid arthritis. 

## Methods

This systematic review was conducted according to a pre-specified protocol (PROSPERO: CRD42021251960) and is reported following the Preferred Reporting Items for Systematic Reviews and Meta-Analyses (PRISMA) statement ^13^.

### Eligibility criteria

Criteria for inclusion, defined according to PECOS (Population, Exposure, Comparator, Outcome, Setting and Study design) ^14^, including outcomes of interest are detailed in Table 1. Criteria were deliberately broad in terms of setting, frailty definition, and outcomes. Briefly, studies must include adults (≥18 years) with rheumatoid arthritis and assess frailty, although we expect studies may mainly involve ‘older’ populations. Studies were considered regardless of frailty measure, to allow comparison between different methods of identifying frailty. We included studies in any setting (community, outpatient, or inpatient). Observational studies with cross-sectional or cohort designs were eligible for inclusion. When examining the association between frailty and clinical outcomes in those with rheumatoid arthritis, studies were expected to report the association between frailty and the outcome of interest. As in previous reviews of frailty ^15, 16^, considered studies that describe this either as the association with the presence or absence of frailty or the association between the degree of frailty and the outcome.

**Table 1.** Inclusion Criteria.

PECOS component	Description
Population	Adults (≥ 18 years old) with rheumatoid arthritis
Exposure	Frailty as assessed by any frailty measure
Comparator	People with rheumatoid arthritis not classified as frail
Outcomes	Primary outcome • Frailty prevalence Secondary outcomes: • Mortality • Hospital admission • Major Adverse Cardiovascular Events • Admission to long-term care facility • Quality of life • Fractures • Disease activity (e.g. Disease Activity Score in 28 joints; DAS-28) • Physical impairment or disability (e.g. Health Assessment Questionnaire – Disability Index; HAQ-DI)
Settings	Community (including care home/nursing home) Outpatient clinic Inpatient
Study design	Cross sectional or cohort
Other exclusions	Conference abstracts, letters, review articles, intervention studies, Grey literature. Studies not published in English.

### Information sources and screening

We searched Medline, Embase, Web of Science Core Collection and Scopus databases from 2001 (as this was the date of the original description of the frailty phenotype and frailty index definitions ^6, 7^) to 8 ^th^ April 2021 using a combination of keywords and Medical Subject Headings. The search structure was ‘rheumatoid arthritis’ and ‘frail’. The full search strategy can be found in the Box 1. Two independent reviewers screened all titles and abstracts and assessed full texts of all relevant articles for eligibility. Disagreements were resolved by consensus, involving a third reviewer if necessary. Hand-searching reference lists of relevant articles and forward-citation searching using Web of Science were also used to supplement electronic database searches.

**Box 1. Search Strategy**

   1.     Exp Arthritis, Rheumatoid/

   2.     (felty$ adj2 syndrome).tw

   3.     (caplan$adj2 syndrome).tw

   4.     Rheumatoid nodule.tw

   5.     (Sjogren$ adj2 syndrome).tw

   6.     (sicca adj2 syndrome).tw

   7.     Still$ disease.tw

   8.     (arthritis adj2 rheumat$).tw

   9.     ((rheumatoid or reumatoid or revmatoid or rheumatic or reumatic or revmatic or rheumat$ or reumat$ or revmarthrit$) adj3 (arthrit$ or artrit$ or diseas$ or condition$ or nodule$)).tw.

   10.     Frail$.tw

   11.     Exp Frailty/

   12.     Exp Frail Elderly/

   13.     1 or 2 or 3 or 4 or 5 or 6 or 7 or 8 or 9

   14.     10 or 11 or 12

   15.     13 and 14

### Data extraction and quality assessment

Data were extracted from each of the eligible studies using a piloted data extraction form. Data extracted included details of the published study (publication reference, aim, setting), population (sample eligibility, recruitment method, age and sex), criteria used to define rheumatoid arthritis (e.g. American College of Rheumatology (ACR)/European League Against Rheumatism (EULAR) criteria ^17^, self-report, electronic medical records, etc.), frailty measure, any adaptation of the frailty measure used in the study, prevalence of frailty, and the association between frailty and clinical outcomes. We used a version of the Newcastle-Ottawa tool, previously adapted to assess observational studies of frailty ^15^, to quantify risk of bias (criteria shown in Box 2).

**Box 2. The Newcastle-Ottawa Scale Adaptation for studies assessing the prevalence and impact of frailty**

**1 – Representativeness of the exposed (i.e. frail) cohort**

a) Truly representative (one star)

b) Somewhat representative (one star)

c) Selected group

d) No description of the derivation of the cohort

**2 – Selection of the non-exposed (i.e. non-frail) cohort**

a) Drawn from the same community as the exposed cohort (one star)

b) Drawn from a different source

c) No description of the derivation of the non-exposed cohort

**3 – Ascertainment of exposure**

a) Validated measurement tool for frailty (two stars)

b) Non-validated measurement tool, but the tool is available or described (one star)

c) No description of measurement tool

**4 – Non-respondents**

a) Comparability between respondents and non-respondents’ characteristics is established, and the response rate is satisfactory (one star)

b) The response rate is unsatisfactory, or the comparability between respondents and non-respondents is unsatisfactory

c) No description of the response rate of the characteristics of the responders and non-responders

**5 – Demonstration that outcome of interest was not present at the start of the study**

a) Yes (one star)

b) No

**Comparability:**

**1 – Comparability of the cohorts on the basis of the design or analysis being controlled for confounders**

a) The study controls for age and sex (one star)

b) The study controls for other factors (one star)

c) Cohorts are not comparable on the basis of the design or analysis controlled for confounders

**Outco**mes:

1 – Assessment of outcomes

a) Independent assessment (one star)

b) Record linkage (one star)

c) Self-report

d) No description

e) Other

**2 – Follow-up long enough for outcomes to occur**

a) Yes (one star)

b) No

**3 – Adequacy of follow-up of cohorts**

a) Complete follow-up: all subjects accounted for (one star)

b) Subjects lost to follow-up unlikely to introduce bias – number lost less than or equal to 20% or description of those lost suggested no different from those followed (one star)

c) Follow-up rate less than 80% and no description of those lost

d) No statement

### Synthesis

Findings of the included studies were summarised using a narrative synthesis. Methodological and demographic details of each study, along with quality assessment, were summarised using tables. Prevalence estimates were plotted stratified by age-group of the sample and with reference to the frailty measure used for each estimate. Findings related to other outcomes (characteristics of rheumatoid arthritis or clinical outcomes) were summarised using a Harvest plot ^18, 19^. Harvest plots can be used to display heterogenous data across a range of outcomes. Findings are displayed on a matrix with each bar representing a study. The position of the bar on the matrix indicates the relationship between frailty and a specific outcome (i.e. positive association, negative association, or no association with frailty status), with the height of the bar indicating the sample size of the study and the colour indicating the frailty measure used.

## Results

**Figure 1.**
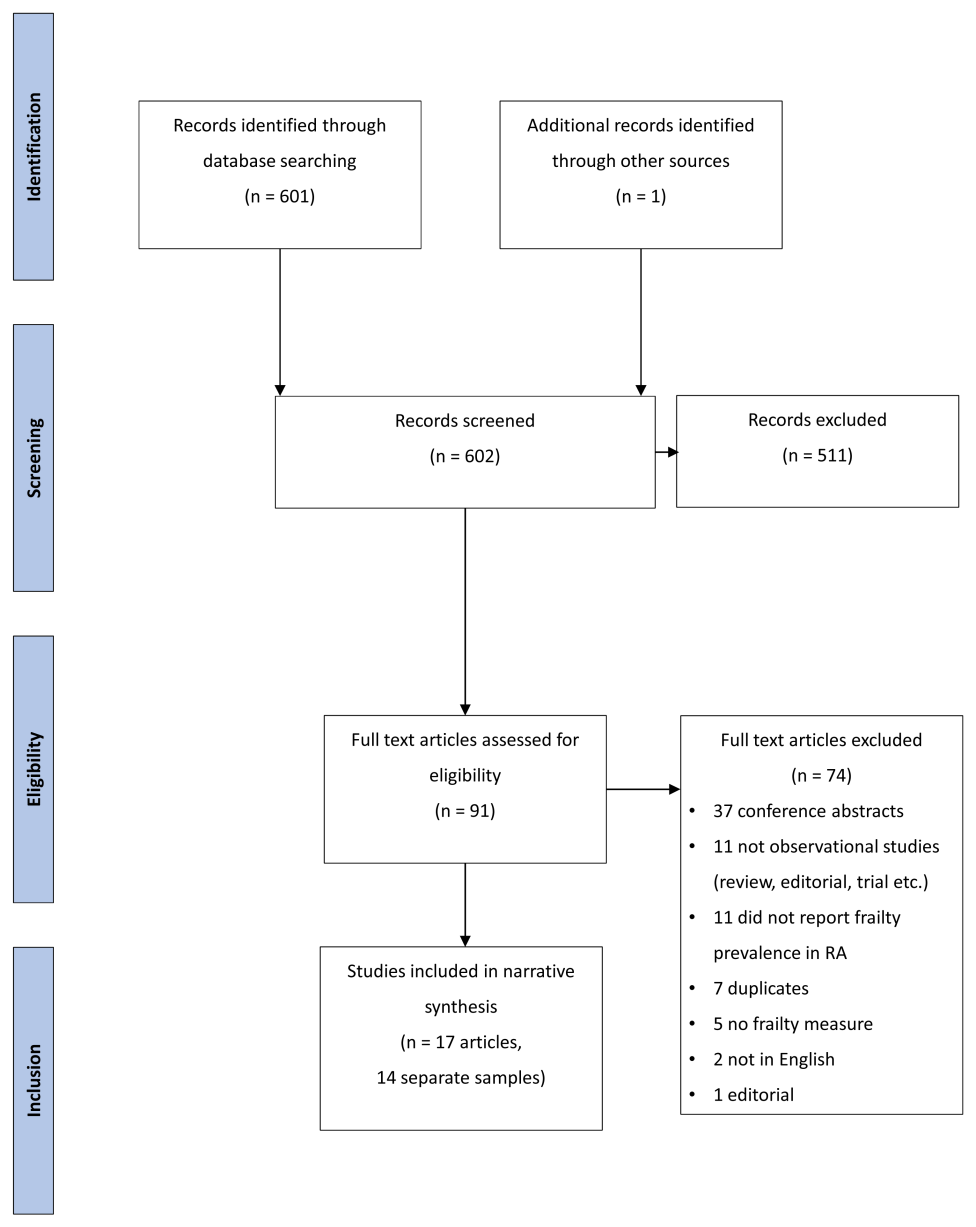
PRISMA diagram of study selection.

Databases searches identified 367 titles and abstracts, after removal of duplicates, of which 91 were retained for full-text screening. From these, 17 eligible full texts were identified, describing 14 separate cohorts (three samples were analysed in two separate papers each). Numbers screened along with reasons for exclusion are shown in Figure 1.

Baseline data for each of the included studies is shown in Table 2. Studies were from Japan (five studies), USA (three studies), Italy, Austria, Canada, Netherlands, Poland and UK (1 study each). Eleven studies identified rheumatoid arthritis according to the 2010 ACR/EULAR criteria ^17^, while others used either ‘clinician diagnosed’ rheumatoid arthritis (three studies), diagnostic codes from primary care records (one study) or did not specify (two studies). Mean age of the study samples ranged from 50.9 to 74.6 years. Only one study presented data on ethnicity ^20^, and none commented on socioeconomic status.

**Table 2.** Characteristics of included studies.

Author, Year	Country	Setting	Frailty measure	Rheumatoid arthritis definition	Total n	Age, years – mean (sd)	N (%) women
Andrews 2017, Andrews 2019 ^12, 21^	USA	Outpatient	Frailty phenotype	ACR	124	58 (10.8)	59 (47.6%)
Bak 2020 ^22^	Poland	Inpatient	Tilburg frailty indicator	ACR/EULAR 2010	106	65.8 (5)	82 (77.4%)
Chang 2010 ^23^	USA	Community	Frailty phenotype	NA	11	74.1 (2.8)	11 (100%)
Haider 2019 ^11^	Austria	Outpatient	SHARE-FI	ACR/EULAR 2010	100	50.9 (9.7)	66 (66%)
Hippisley-Cox 2017 ^24^	UK	Community	Qfrailty	Primary Care clinical coding	10312	-	-
Kojima 2020 ^25^	Japan	Outpatient	Kihon checklist	ACR 2010	375	65.2 (9.7)	323 (86.1%)
Li 2019 ^26^	Canada	Outpatient (registry)	Frailty index	"Active RA"	2923	57.7 (12.7)	2290 (78.3%)
Minamino 2021 ^27^	Japan	Outpatient	Study of Osteoporotic Fracture frailty indicator	NA	306	63.5	306 (100%)
Oetsma 2020 ^28^	Netherlands	Outpatient	Gronigen frailty indicator, Geriatric 8	rheumatologist diagnosed RA	80	74.6 (5.9)	53 (66.2%)
Salaffi 2019 ^29^ *	Italy	Outpatient	SHARE-FI	ACR/EULAR	210	60.4 (13.5)	138 (65.7%)
Salaffi 2020 ^10^ *	Italy	Outpatient	Comprehensive Rheumatologic Assessment of Frailty	ACR/EULAR	219	60.4 (13.5)	138 (63%)
Tada 2019, Tada 2021 ^30, 31^	Japan	Outpatient	Kihon checklist	ACR/EULAR	95	68 (5.5)	78 (82.1%)
Wysham 2020 ^20^	USA	Outpatient	Frailty phenotype	rheumatologist diagnosed RA	138	58 (10.8)	117 (84.8%)
Yoshii 2019 ^32^	Japan	Outpatient	Frailty phenotype	ACR/EULAR	441	64.5 (13.5)	337 (76.4%)
Yoshii 2020 ^33^	Japan	Outpatient	5-item frailty score	ACR/EULAR	739	71.3	-

*Based on same sample, presented on separate lines as report different frailty measure. ACR: American College of Rheumatology, EULAR: European Alliance of Associations of Rheumatology, RA: rheumatoid arthritis SHARE-FI: Survey for Health, Aging and Retirement in Europe Frailty Instrument. UK: United Kingdom, USA: United States of America

The quality assessment of the included studies is summarized in Table 4. Most samples were recruited from rheumatology clinics, with most judged to be representative based on subsequent exclusion criteria and sampling methods. Frailty measures used were generally validated or well-described. Few studies presented data on non-responders.

### Frailty measurement

Across the 14 included studies, 11 different frailty measures were used. These are summarised in Table 3. The most commonly used measure was the frailty phenotype described by Fried *et al.* (five studies, six papers), followed by the Kihon frailty checklist (two studies, three papers) and the SHARE frailty instrument (an adaptation of the frailty phenotype developed from the Survey for Health, Aging and Retirement in Europe, reported in two studies).

**Table 3.** Frailty measures used in included studies.

Frailty measure	Components	Range and categorisation	Outcomes reported in included studies	Included studies
Frailty phenotype ^6^	5 components (unintentional weight loss, exhaustion, low grip strength, slow walking pace, low physical activity)	1-2 criteria: Pre-frail ≥3 criteria: Frail	Frailty prevalence, Duration of rheumatoid arthritis, Disease activity, HAQ-DI	Andrews 2017 ^12^, Andrew 2019 ^21^, Chang 2010 ^23^, Wysham 2020 ^20^, Yoshii 2019 ^32^
Kihon checklist ^34^	Self-administered checklist (components: activities of daily living, exercise, falling, nutrition, oral health, cognition, depression)	Unweighted sum of components. Range 0–25. Pre-frail (4–7), Frail (≥8).	Frailty prevalence, Duration of rheumatoid arthritis, Disease activity, HAQ-DI	Kojima 2020 ^25^, Tada 2019 ^30^, Tada 2021 ^31^
Survey for Health, Aging and Retirement in Europe Frailty Instrument (SHARE-FI) ^35^	5 components (unintentional weight loss, exhaustion, low grip strength, slow walking pace, low physical activity). Conceptually based on the frailty phenotype with an alternative calculation for the final categorisation of frailty.	Weighted score calculated and then categorised into robust, pre- frail, frail.	Frailty prevalence, Disease activity, HAQ-DI	Haider 2019 ^11^, Salaffi 2019 ^29^
Frailty index ^7, 8^	Count of health-related deficits (≥30, type and number of chosen deficits may vary between studies). Total present divided by number of possible deficits	Range 0-1 Sometimes categorised (threshold for frailty varies (e.g. 0.2, 0.24)	Hospitalisation, Fractures	Li 2019 ^26^
Comprehensive Rheumatologic Assessment of Frailty (CRAF) ^10^	10 domains identified as relevant to the assessment of frailty in the context of rheumatological condition. Conceptually similar to the frailty index, cumulative deficit model (but with fewer deficits than the frailty index).	Range 0-1 Authors propose to categorise as robust (0–0.12), mild (0.12–0.24), moderate (0.24–0.36) and severe (>0.36) frailty.	Frailty prevalence, disease activity	Salaffi 2020 ^10^
5-Item frailty risk score ^36^	5 components (weight loss, fatigue, short term memory decline, slow walking pace, low physical activity). Conceptually based on the frailty phenotype, with alteration of variables included.	1-2 criteria: Pre-frail ≥3 criteria: Frail	Frailty prevalence, Disease activity, HAQ-DI	Yoshii 2020 ^33^
Tilburg frailty indicator ^37^	15 questions across 3 domains (physical, psychological and social) Responses combined into unweighted sum.	Range 0–15 ≥5 indicates frailty	Frailty prevalence	Bak 2020 ^22^
Geriatric 8 score ^38^	8 domains scored and summed (nutritional status, weight loss, body mass index, motor skills, psychological, number of medications, self-rated health, age)	Range 0–17 <14 indicates frailty	Frailty prevalence	Oetsma 2020 ^28^
Groningen frailty indicator ^39^	15 items across 4 domains (physical, cognitive, social and psychological).	Range 0–15 ≥4 indicates frailty	Frailty prevalence, HAQ-DI	Oetsma 2020 ^28^
Study of Osteoporotic Fracture frailty indicator	3 components (weight loss, chair stand, exhaustion)	1 component: prefrail 2-3 components: frail	Frailty prevalence, Duration of rheumatoid arthritis, Disease activity, HAQ-DI	Minamino 2021 ^27^
QFrailty ^24^	Algorithm based on electronic medical records combining mortality (QMortality score) and hospital admission (QAdmission score) risk.	Categorised as mild, moderate and severe frailty.	Frailty prevalence	Hippisley-Cox 2017 ^24^

Table adapted from Hanlon *et al*. 2020 ^15^

**Table 4.** Quality assessment of included studies (based on adapted Newcastle Ottawa Scale).

Author, Year	Representative	Selection of non frail comparison	Ascertainment of exposure frailty	Non respondents	Outcome not present at start	Controls for age and sex	Control for other factors	Outcome assessment	Length of follow up	Adequacy of follow up	Cross-sectional score	Longitudinal score
Andrews 2017, Andrews 2019 ^12, 21^	1	1	2	0	1	1	1	1	1	1	4/5	10/11
Bak 2020 ^22^	0	1	2	0	-	-	-	-	-	-	3/5	-
Chang 2010 ^23^	1	1	1	0	-	-	-	-	-	-	3/5	-
Haider 2019 ^11^	1	1	2	0	-	-	-	-	-	-	3/5	-
Hippisley-Cox 2017 ^24^	1	1	1	0	-	-	-	-	-	-	3/5	-
Kojima 2020 ^25^	0	1	2	0	-	-	-	-	-	-	3/5	-
Li 2019 ^26^	1	1	2	0	1	1	1	1	1	1	4/5	10/11
Minamino 2021 ^27^	1	1	2	0	-	-	-	-	-	-	4/5	-
Oetsma 2020 ^28^	1	1	2	0	-	-	-	-	-	-	4/5	-
Salaffi 2019 ^29^	1	1	2	1	-	-	-	-	-	-	5/5	-
Salaffi 2020 ^10^	1	1	1	1	-	-	-	-	-	-	4/5	-
Tada 2019, Tada 2021 ^30, 31^	1	1	2	1	-	-	-	-	-	-	4/5	-
Wysham 2020 ^20^	1	1	2	0	-	-	-	-	-	-	4/5	-
Yoshii 2019 ^32^	1	1	2	0	-	-	-	-	-	-	4/5	-
Yoshii 2020 ^33^	1	1	1	0	-	-	-	-	-	-	3/5	-

Of the five studies that used the frailty phenotype (based on grip strength, weight loss, physical activity, exhaustion, and walking speed), two also explored alternatives to grip strength, given the potential for the measurement of grip strength to be impacted by rheumatoid arthritis affecting the hands. Both used lower extremity strength as an alternative to grip strength to capture ‘weakness’.

### Frailty prevalence

**Figure 2.**
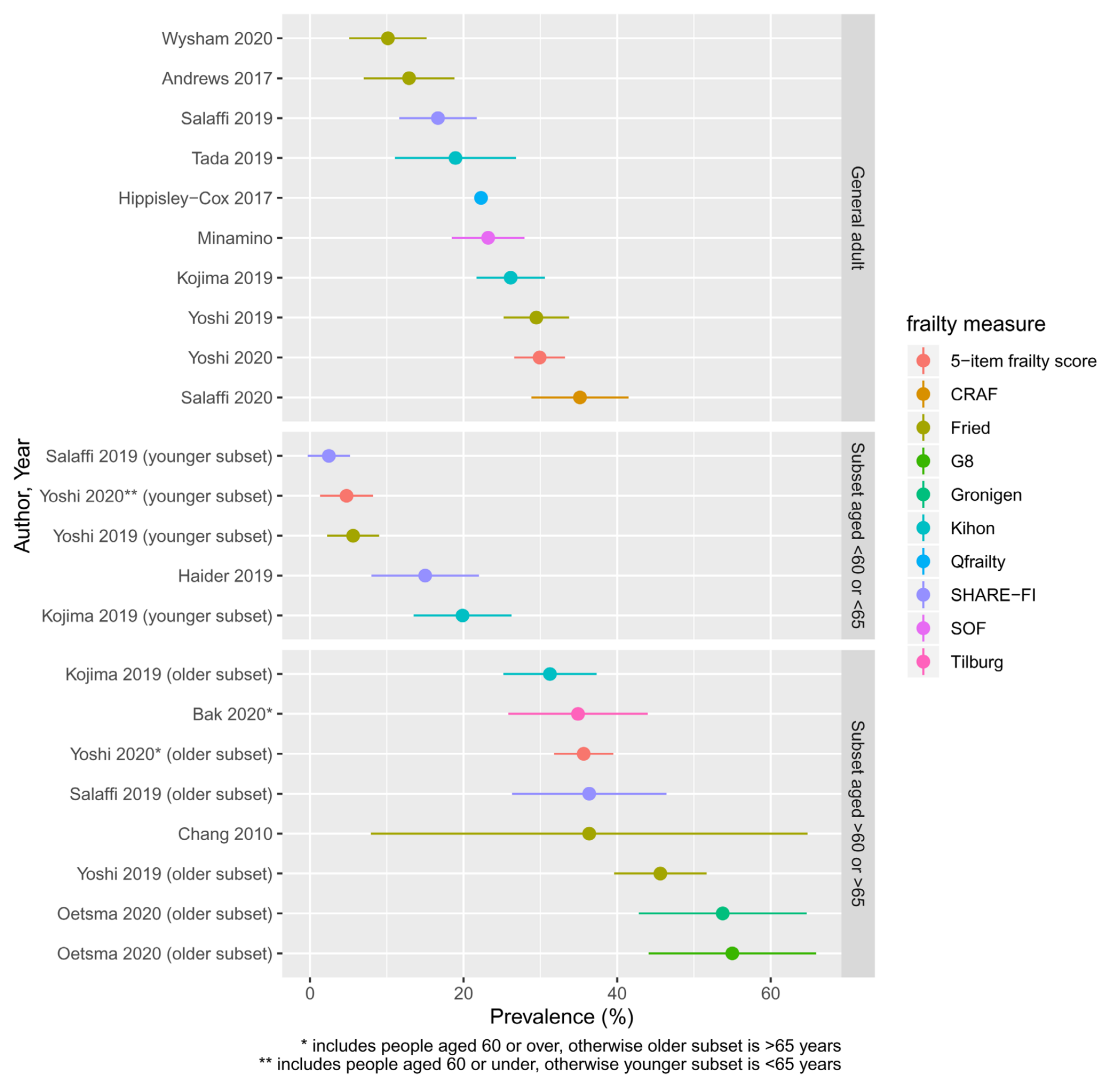
Prevalence of frailty. Colours indicate frailty measure. Points indicate point estimate of for frailty prevalence, with bars indicating 95% confidence intervals. Ordered by frailty prevalence for ease of comparison.

The prevalence of frailty in each of the studies identified is shown in Figure 2, stratified by age group. The prevalence in general adult populations with rheumatoid arthritis ranged from 10.1% (using the frailty phenotype) to 36% (using the Comprehensive Rheumatologic Assessment of Frailty (CRAF), taking ‘moderate frailty’ as the cut-off). Studies (or subsets of studies) with populations aged under 60 or 65 years had a frailty prevalence ranging from 2.4% (frailty phenotype) to 19.9% (Kihon checklist). In older populations, estimates ranged from 31.2% (Kihon checklist) to 55% (Geriatric 8 tool).

While frailty prevalence is recognised to vary depending on the measure used, and therefore heterogeneity in these estimates is expected, the prevalence of frailty varied widely even among similar frailty definitions. For example, three studies applied the frailty phenotype to general adult populations with prevalence estimates of 10.1%, 12.9% and 28.5%, respectively. Two studies applied the SHARE-FI to populations aged under 65 years and found a prevalence of 2.5% and 15%, respectively. Therefore, estimates of frailty prevalence in rheumatoid arthritis appear to vary widely even between samples of similar ages applying similar measures of frailty.

One study assessed frailty using the standard frailty phenotype definition, and then using an alternative measure of weakness based on lower extremity strength rather than grip. This was to limit the impact of rheumatoid arthritis affecting the hands on the assessment of frailty. The prevalence of frailty using this alternative strength assessment was lower than the standard grip strength assessment (3.6% and 12.9%, respectively).

We did not attempt to meta-analyse any estimates of frailty prevalence as it is not valid to directly compare frailty prevalence assessed by different measures, and, even for those studies using similar measures, population demographics and exclusion criteria were too heterogenous to allow for a meaningful estimate.

### Relationship between frailty and clinical characteristics and outcomes

**Figure 3.**
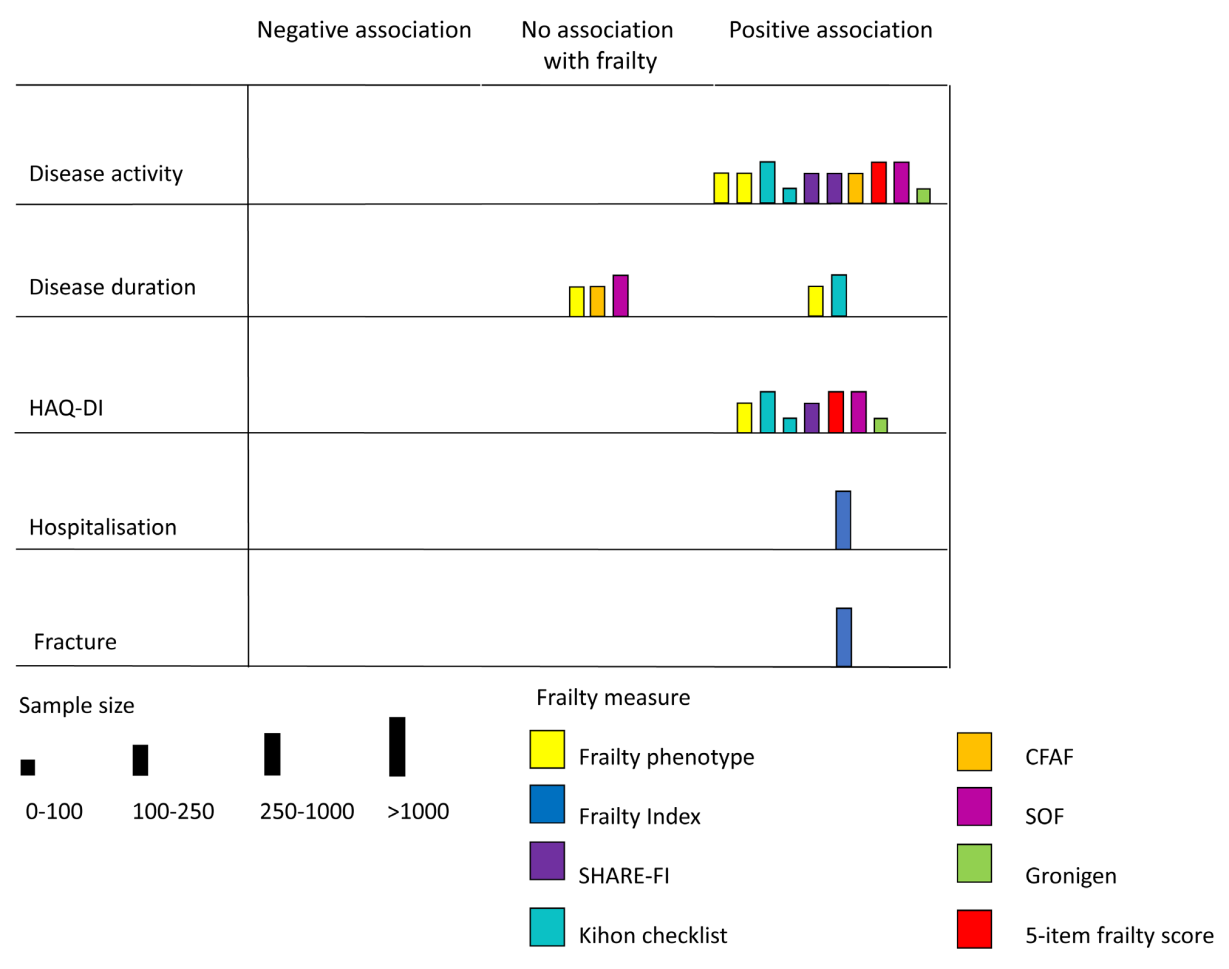
Association between frailty and clinical outcomes. Each bar represents a study. The position of the bar on the matrix indicates the association between frailty and the outcome in question (positive association, negative associaton or neutral assocaiton). Colour indicates the frailty measure used in the study. The weight of the bar indicates the study sample size.

Associations between frailty and clinical characteristics or outcomes in rheumatoid arthritis are summarised in Figure 3. Most (8/10) of these studies were cross-sectional, showing associations between frailty and baseline measures of disease activity or physical function. These are discussed in greater detail below.

***Rheumatoid arthritis disease activity.*** Ten studies, using seven different frailty measures and four different markers of rheumatoid arthritis disease activity (four using Disease Activity Score in 28 joints, two using the Rheumatoid Arthritis Disease Activity Index, two using Simple Disease Activity Index and two using Clinical Disease Activity Index) all showed a significant cross-sectional association between frailty status and activity of rheumatoid arthritis before adjustment for additional factors ^10, 11, 20, 21, 25, 27– 30, 33^. One study, using CDAI, found that this relationship was no longer evident after adjusting for age ^33^. In contrast, two other studies, showed that frailty remained associated with a higher baseline DAS-28 score after adjustment for age, sex, duration of rheumatoid arthritis and physical impairment (quantified using the Health Assessment Questionnaire – Disability Index (HAQ-DI)) ^25, 27^.

Two studies presented data on prevalence or degree of frailty, stratified by disease activity (remission, low, medium or high). Tada and colleagues assessed frailty using the Kihon checklist and reported a prevalence of 6.7% in the remission group, 18% in people with low disease activity, and 47% in the medium or high disease activity group ^30^. Salaffi and colleagues, analysing the CRAF, showed that none of the participants in remission or with low disease activity groups had scores above the threshold for ‘moderate frailty’, whereas among participants with high disease activity the median CRAF score was 0.34 (close to the threshold for ‘severe frailty’ of 0.36) ^10^.

One cohort study assessed the relationship between frailty and change in disease activity over time, reporting no significant association between frailty and change in RADAI over 3.7 years follow-up ^21^.

Taken together these data show a consistent relationship between frailty and disease activity, assessed by a diverse range of measures. The prevalence of frailty appears considerably higher in people with active disease. However, these were cross sectional assessments and no studies assessed whether frailty prevalence or severity is sensitive to changes in disease severity over time.

***Physical function.*** Seven studies assessed the relationship between frailty and physical function using the HAQ-DI ^11, 12, 21, 22, 25, 27, 28, 30, 33^. Each of these studies demonstrates an association between frailty and higher baseline HAQ scores (indicating a greater degree of physical impairment). One of these studies also included a longitudinal analysis in which frailty at baseline (assessed using the frailty phenotype) was associated with worsening of HAQ scores over two-years follow-up, indicating that participants with frailty at baseline were more likely to experience deterioration in physical function than robust participants ^21^. This analysis was also adjusted for rheumatoid arthritis disease activity. Together these findings show a consistent relationship between frailty status, assessed through a range of measures, and greater physical impairment assessed using HAQ.

***Duration of rheumatoid arthritis.*** Five studies assessed the relationship between frailty and the duration of rheumatoid arthritis at baseline ^10, 20, 25, 27, 29^. Findings were mixed, with three studies showing no association between frailty and disease duration ^10, 20, 27^. By contrast, two studies showed that frailty was associated with greater duration of rheumatoid arthritis at the time of assessment ^25, 29^, however only one of these studies additionally adjusted for age in the analysis ^25^.

***Other outcomes.*** One study, using the frailty index approach to quantifying frailty in 2923 participants, assessed the relationship between frailty and all-cause hospitalisations ^26^. Higher frailty index values were associated with a greater risk of hospitalisation during a mean follow-up of 3.7 years. This same study also showed that a higher frailty index was associated with a greater risk of osteoporotic fractures over the same follow-up period.

No studies assessed the relationship between frailty and mortality, cardiovascular events, or outcomes in response to treatment. Also, no studies assessed frailty at any other time-points following baseline, and therefore no analyses were identified of frailty trajectories in rheumatoid arthritis or of factors associated with worsening or amelioration of frailty.

## Discussion

### Summary of findings

In this systematic review we identified 17 papers, based on 14 different populations, reporting the prevalence of frailty in people with rheumatoid arthritis. Frailty was common in all studies, ranging from 10% to 36% among adult populations with rheumatoid arthritis, however there was considerable heterogeneity in both the measures used to identify frailty and the demographics of the populations studied (most notably age). There were 11 different measures used to identify frailty across the 14 cohorts, which limits the comparability of prevalence estimates. However, even among studies using similar measures, estimates of the frailty prevalence were variable. This may reflect differences in the underlying population (e.g. ethnicity, socioeconomic status, disease activity), inclusion criteria, or the application of frailty measures. It is notable, therefore, that few studies reported data on ethnicity or socioeconomic status.

Nonetheless, frailty (however measured) was consistently associated with greater disease activity assessed through scores such as DAS-28, and with greater physical impairment indicated by HAQ-DI. The relationship with duration of rheumatoid arthritis was inconsistent, with some studies reporting an association between frailty and greater duration of rheumatoid arthritis. None assessed the prevalence of frailty in new-onset rheumatoid arthritis. Most studies were cross sectional, with only two reporting longitudinal follow-up (showing frailty to be associated with hospitalisations and fractures, and worsening physical function, respectively). Therefore, the prognostic significance of frailty in rheumatoid arthritis remains unclear, nor do we know anything about the likely trajectory of frailty over time or the sensitivity of frailty to changes in disease activity as a result of treatment with disease-modifying antirheumatic drugs.

### Findings in context of previous literature

Estimates of frailty prevalence are understood to be limited by variability in how frailty is measured. Different frailty measures are based on different characteristics, are underpinned by different theoretical constructs, and identify different populations. A recent systematic review estimated a pooled global prevalence of frailty in the general population at 7% (95% CI 5-9%) using a physical frailty model and 24% (22-26%) using a cumulative deficit model, however estimates vary widely depending on the underlying population demographics ^40^. Despite these limitations in comparing frailty prevalence between studies, the estimates reported in this review indicate that frailty is common in people with rheumatoid arthritis compared to the general population. This is consistent with previous observations that frailty, identified using a frailty index, was common in phase 3-4 randomised controlled trials of people with rheumatoid arthritis ^41^. As in this review, frailty in these trials was strongly associated with greater disease activity.

The cross-sectional nature of the included studies makes determining the extent to which the frailty is caused by rheumatoid arthritis difficult. The development of frailty is understood to be multifactorial. Furthermore, different approaches to identifying frailty (such as a frailty phenotype versus a cumulative deficit model, or a physical model versus one including psychological and social vulnerability) may have different causal pathways and mechanisms underlying them ^42, 43^. However, rheumatoid arthritis may lead to a range of states or complications (such as fatigue, sarcopenia, weight loss, and functional limitation) which may all contribute to the identification of frailty. Fatigue in rheumatoid arthritis may result from the underlying inflammatory process as well as symptoms, functional, emotional and psychological impact of the condition and treatments ^44^. Weight loss and low body mass index, thought partly to be mediated through excess pro-inflammatory mediators such as IL-1 and TNF-alpha, are associated with greater erosive disease in rheumatoid arthritis as well as greater cardiovascular risk, physical disability, and mortality ^45– 47^. Rheumatoid arthritis, through a combination of systemic inflammation and reduced physical activity, may also result in sarcopenia which in turn contributes to the development of frailty ^48– 52^. These observations, along with the consistent association between frailty and greater disease activity, mean it is likely that rheumatoid arthritis – particularly if highly active or severe – leads to the development of features of frailty.

Conversely, frailty has a wide range of potential causes and associations, and it is unlikely that there is a single common pathway or mechanism underlying the development of frailty in people with rheumatoid arthritis. Co-existing frailty alongside rheumatoid arthritis may lead or contribute to functional limitations not exclusively attributable to rheumatoid arthritis itself. The rationale for frailty identification and assessment is to facilitate a broad and multidimensional evaluation of a person’s needs and priorities. Given increasing rheumatoid arthritis in older age ^1^, and the prevalence of multimorbidity among people with rheumatoid arthritis ^53^, it is important to better understand the whether incporporating frailty assessment into the management of rheumatoid arthritis would being additional benefits beyond those measures already commonly used.

### Implications

These findings highlight several important gaps in our understanding of frailty in the context of rheumatoid arthritis. The first is the prognostic significance of frailty in people with rheumatoid arthritis. Only one study, using a frailty index model, assessed the association between frailty and hospitalisations and none explored whether frailty is associated with mortality, cardiovascular events, or long-term care needs in people with rheumatoid arthritis. The association between frailty and these outcomes in the general population is well established. However, given the overlap between features of active or severe rheumatoid arthritis and frailty, it is not clear if assessment of frailty in the context of rheumatoid arthritis improves prediction of these outcomes.

The second gap is to disentangle the relationship between frailty and rheumatoid arthritis disease activity. Active rheumatoid arthritis may give rise to a range of features which may indicate frailty (fatigue, weakness, pain, functional limitation, etc.). Frailty may, therefore, be amenable to intervention. Frailty is recognised to be a dynamic state which changes over time. However, the degree to which frailty in rheumatoid arthritis is reversible is not clear. This question, like the association between frailty and clinical outcomes, would require longitudinal studies ideally with serial assessments of both frailty and disease activity.

A final, more nuanced, gap in our understanding is how these epidemiological measures of frailty translate to the experience and understanding of people living with rheumatoid arthritis and to the clinical impression of professionals involved in their care. While a range of physical, functional, and psychological features common in rheumatoid arthritis may be consistent with current definitions of frailty, this may not be how people living with rheumatoid arthritis would choose to characterise their experience. It is also not clear if frailty identified in such a way, particularly when it results from active rheumatoid arthritis, is equivalent to frailty as it would be understood by clinicians. Understanding the implications of frailty in rheumatoid arthritis therefore not only requires a fuller understanding of its epidemiology, but also the broader clinical implications and the utility of a frailty ‘label’.

### Strengths and limitations

Strengths of this review include a comprehensive search strategy with duplicate screening and data extraction. However, the search was limited to English language only and we excluded Grey literature. This could potentially lead to language or publication bias, respectively. It was not possible to conduct a meta-analysis of frailty prevalence due to the degree of heterogeneity. This was particularly evident in the measurement of frailty, as a range of different measures were used and prevalence estimates are therefore not directly comparable. Studies were also heterogenous in terms of their inclusion criteria, demographics, and definitions of rheumatoid arthritis. Studies were all from high-income countries with no data from upper-middle income of LMICs. Also only one study presented data on the ethnicity of participants, and none assessed socioeconomic status, factors which may impact the prevalence of frailty. Finally, the studies included in this review were observational and mostly cross-sectional. It is therefore not possible to assess causal relationships.

## Conclusion

Frailty in people with rheumatoid arthritis has been quantified in high income countries using a wide range of different approaches and is consistently demonstrated to be common, particularly among people with more active disease. Assessment of frailty among people with rheumatoid arthritis, including those aged under 65 years, is likely to identify people at greater risk of functional limitation. However, a relative lack of longitudinal studies and heterogeneity in the methods used to assess frailty mean that the clinical implications, prognostic significance, and potential reversibility remain unclear. There is a need for studies in upper-middle-income countries or LMICs as well as studies with serial follow-up and repeated measures to understand the trajectories and outcomes of frailty in rheumatoid arthritis, as well as greater exploration of the implications of frailty from the perspective of patients and clinicians. Understanding these relationships in greater detail may reveal potential for interventions to ameliorate frailty in rheumatoid arthritis, limit its impact, and support people living with frailty.

## Data availability

### Underlying data

Zenodo: Data underlying Frailty in people with rheumatoid arthritis – A systematic review of observational studies, https://doi.org/10.5281/zenodo.5515860 ^54^.

### Reporting guidelines

PRISMA checklist available at Zenodo: Data underlying Frailty in people with rheumatoid arthritis – A systematic review of observational studies, https://doi.org/10.5281/zenodo.5515860 ^54^.

Data are available under the terms of the Creative Commons Attribution 4.0 International license (CC-BY 4.0).
