# Supplementary material for: Frailty in people with rheumatoid arthritis: a systematic review of observational studies
Source: Wellcome Open Res. 2022 Sep 20;6:244. Originally published 2021 Sep 23. [Version 2] doi: 10.12688/wellcomeopenres.17208.2 (PMC10511856; doi:10.12688/wellcomeopenres.17208.2)
Supplement: Peer review history

# Reviewer report: Reviewer response for version 2

**DOI:** 10.21956/wellcomeopenres.20165.r60599

**Published:** 2023-09-20

**Authors:** Yasumori Sobue

**Affiliations:** 1 Japan Red Cross, Aichi Medical Center, Nagoya University, Nagoya Daiichi Hospital, Michishita-cho, Nagoya, Japan

This study aims to investigate a systematic review of observational studies on frailty in people with rheumatoid arthritis. As the authors have mentioned, due to heterogeneity of frailty measures or evaluations of factors, the significance of a systematic review without meta-analysis is uncertain. However, in this study, they successfully visually represented them using different colors, even though it might be impractical to group different measures together.

 There are minor points that need to be improved.

- Abstract: these is heterogeneity → these are heterogeneity
- Figure 1: Can you show the content of “Records excluded (n=1274)”? But is it n=511(601-90)? “Studies included in review (n=17)” and “Reports of included studies (n=17)” seem to have 34, but they are not.
- Figure 3 was difficult to see. Would you put "Sample size" or "Frailty measure" in the right space?

Are the rationale for, and objectives of, the Systematic Review clearly stated?

Partly

Is the statistical analysis and its interpretation appropriate?

Yes

Are sufficient details of the methods and analysis provided to allow replication by others?

Yes

Are the conclusions drawn adequately supported by the results presented in the review?

Yes

Reviewer Expertise:

Rheumatoid arthritis, frailty, locomotive syndrome

I confirm that I have read this submission and believe that I have an appropriate level of expertise to confirm that it is of an acceptable scientific standard.

# Reviewer report: Reviewer response for version 2

**DOI:** 10.21956/wellcomeopenres.20165.r52462

**Published:** 2022-11-01

**Authors:** Dhammika Deepani Siriwardhana

**Affiliations:** 1 Research Department of Primary Care and Population Health, University College London, London, UK

The authors have addressed my comments adequately. I am happy to approve the manuscript for indexing.

Are the rationale for, and objectives of, the Systematic Review clearly stated?

Partly

Is the statistical analysis and its interpretation appropriate?

Yes

Are sufficient details of the methods and analysis provided to allow replication by others?

Partly

Are the conclusions drawn adequately supported by the results presented in the review?

Yes

Reviewer Expertise:

Public Health, Epidemiology

I confirm that I have read this submission and believe that I have an appropriate level of expertise to confirm that it is of an acceptable scientific standard.

# Reviewer report: Reviewer response for version 1

**DOI:** 10.21956/wellcomeopenres.19016.r50113

**Published:** 2022-06-20

**Authors:** Rose Galvin

**Affiliations:** 1 Ageing Research Centre, Health Research Institute, University of Limerick, Limerick, Ireland

Many thanks for inviting me to review this systematic review of cohort studies exploring the prevalence of frailty and its association with clinical outcomes among people with RA. I have included some comments that would help in the reading and interpretation of the findings:

**Abstract**

Background

- State the clinical outcomes that you are interested in. Process outcomes are also reported in the findings.

Methods

- What approach was used to diagnose frailty?
- What approach was used to synthesise the data?
- Was study quality also assessed independently?

**Results**

- It would be helpful if the timeframe between exposure and outcome was quantified for each outcome.

**Introduction**

- The introduction quantifies the burden of the problem of RA and associated morbidities. However, the rationale for conducting the SR warrants further consideration. Why is it important to recognise frailty in this population in terms of risk stratification and subsequent allocation of resources?
- The differences between objectives 3 and 4 are unclear. You have also reported findings across other outcomes (e.g. hospitalisaton) other than those reported in objectives. Please correct this inconsistency.

**Methods**

- How was the PRIMSA statement modified to inform the conduct and reporting of this study? Consider using the MOOSE guidelines and the PRISMA flow diagram.
- What definitions of RA are eligible for inclusion?
- The authors mention that ‘Studies were considered regardless of frailty measure, to allow comparison between different methods of identifying frailty’. Were validated measures of frailty considered only or studies where self-reporting or clinical judgment was used to diagnose frailty in the absence of any set criteria?
- Study designs – was the control arm of experimental studies considered for inclusion?
- Was an informational specialist involved in developing the search string?
- Check consistency in reporting the number of databases searched in abstract/methods.
- Insert initials of reviewers involved in screening, full-text selection, data extraction, and quality appraisal.
- Perhaps include a rationale for why the Newcastle-Ottawa tool was chosen as an RoB tool.
- How was the variation in the reporting of adverse outcomes handled (for example, the differences in reporting functional decline across studies). Greater clarity is needed on the parameters used to extract data.

**Results**

- Overall, the findings to map the objectives of the review are clearly reported.
- It would be helpful to include more details in the narrative on the impact of the quality appraisal on the interpretation of findings.

**Discussion**

- Consideration needs to be given to other factors that might influence the prevalence of frailty aside from age and the measure of frailty?
- The results are well contextualised with regard to the current literature. The strengths and weaknesses are well-considered.
- Perhaps insert a few lines on the clinical implications of frailty in this population for a clinical audience.

Are the rationale for, and objectives of, the Systematic Review clearly stated?

Yes

Is the statistical analysis and its interpretation appropriate?

Partly

Are sufficient details of the methods and analysis provided to allow replication by others?

Partly

Are the conclusions drawn adequately supported by the results presented in the review?

Yes

Reviewer Expertise:

Ageing, quantitative research methods

I confirm that I have read this submission and believe that I have an appropriate level of expertise to confirm that it is of an acceptable scientific standard, however I have significant reservations, as outlined above.

# Response

**Published:** 2022-08-05

**Authors:** Peter Hanlon

**Affiliations:** University of Glasgow, UK

Thank you for your detailed review of our manuscript. We have now uploaded a revised version based on your comments and those of the other reviewer. Please find below our response to each of the comments. Thank you again for your time and consideration in reviewing our manuscript.

 Review 2:

 Many thanks for inviting me to review this systematic review of cohort studies exploring the prevalence of frailty and its association with clinical outcomes among people with RA. I have included some comments that would help in the reading and interpretation of the findings:

**Abstract**

Background

- State the clinical outcomes that you are interested in. Process outcomes are also reported in the findings.

We have edited the following sentences: “We undertook a systematic review to assess prevalence of frailty in people with rheumatoid arthritis, and the relationship between frailty and disease activity or clinical outcomes” and “[we searched for studies]… analysing the relationship between frailty and disease activity or clinical outcomes (e.g. quality of life, hospitalisation or mortality) in people with rheumatoid arthritis”.

Methods

- What approach was used to diagnose frailty?

**Author response: **We have added text to the abstract to specify that we would consider “any frailty measure”.

  

- What approach was used to synthesise the data?

**Author response:** We have now added: “We used narrative synthesis”.

  

- Was study quality also assessed independently?

**Author response: **Yes, these were. We have added the following text: “Screening, quality assessment and data extraction were performed independently by two reviewers.”

**Results**

- It would be helpful if the timeframe between exposure and outcome was quantified for each outcome.

**Author response:** We have edited this sentence in the abstract to present this information: “Frailty was cross-sectionally associated with higher disease activity (10/10 studies), lower physical function (7/7 studies) and longer disease duration (2/5 studies), and with hospitalization and osteoporotic fractures (1/1 study, 3.7 years follow-up).”

**Introduction**

- The introduction quantifies the burden of the problem of RA and associated morbidities. However, the rationale for conducting the SR warrants further consideration. Why is it important to recognise frailty in this population in terms of risk stratification and subsequent allocation of resources?

**Author response: **We have added the following text: “This is important as frailty measures are increasingly being advocated to aid risk stratification and identification of high-risk populations in a range of clinical contexts. The utility and appropriateness of such an approach in people with rheumatoid arthritis therefore requires careful consideration of the relationship between frailty and this condition.”

  

- The differences between objectives 3 and 4 are unclear. You have also reported findings across other outcomes (e.g. hospitalisaton) other than those reported in objectives. Please correct this inconsistency.

**Author response**: We have re-worded the final aim in the background section to make specific reference to the types of outcomes we are interested in: “what is the association between frailty and adverse health outcomes (e.g. hospitalisation, mortality or quality of life) in people with rheumatoid arthritis.” The full list of outcomes is given in Table 1.

**Methods**

- How was the PRIMSA statement modified to inform the conduct and reporting of this study? Consider using the MOOSE guidelines and the PRISMA flow diagram.

**Author response:** Thank you. We have added the MOOSE checklist to the supplementary file.

  

- What definitions of RA are eligible for inclusion?

**Author response.** We have added the following to the methods to clarify our inclusion criteria “We did not exclude studies on the basis of the criteria used to define rheumatoid arthritis (i.e. validated criteria, physician diagnosis, medical record/clinical codes or self-reported definitions were all eligible for inclusion).”

  

- The authors mention that ‘Studies were considered regardless of frailty measure, to allow comparison between different methods of identifying frailty’. Were validated measures of frailty considered only or studies where self-reporting or clinical judgment was used to diagnose frailty in the absence of any set criteria?

**Author response: **We have added the following text to clarify the criteria used to determine eleigibility of a frailty measure: “These could include validated measures of frailty (e.g. frailty phenotype or frailty index), adaptations of these measures where the adaptation was described, or unvalidated measures intended to capture frailty as long as the criteria used to define frailty within the study were fully described.”

  

- Study designs – was the control arm of experimental studies considered for inclusion?

**Author response: **Experimental studies were not part of our inclusion criteria (although we accept the reviewer’s point that the control arm could be a useful source of information should such studies have been identified).

  

- Was an informational specialist involved in developing the search string?

**Author response:** For this search strategy we did not involve a specialist, although the search terms for rheumatoid arthritis were taken from previous reviews in which a research librarian informed the development of the search strategy.

  

- Check consistency in reporting the number of databases searched in abstract/methods.

**Author response: **Thank you for highlighting this. We have amended the abstract to state four databases were searched.

  

- Insert initials of reviewers involved in screening, full-text selection, data extraction, and quality appraisal.

**Author response: **We have added this information to the text.

  

- Perhaps include a rationale for why the Newcastle-Ottawa tool was chosen as an RoB tool.

**Author response: **We have added the following text: “The Newcastle-Ottawa scale is frequently used to assess quality of observational studies. Previous reviews have also adapted elements of the scale to reflect the studies of interest to the review itself. For this review, we used an adaptation previously developed for observational studies of frailty. This adaptation altered the ‘exposure’ component of the was altered to award two points if a study used validated measure of frailty implemented according to its original description. One point was awarded if studies used an alternative measure of frailty (e.g. an adapted or non-validated measure of frailty) but the criteria were described in sufficient detail to allow the assessment to be replicated. This adaptation was to reflect the fact that there is no ‘gold-standard’ measure of frailty and that frailty is assessed using a diverse range of measures within the literature. The scale was applied to all studies (cross sectional or longitudinal), with only the first 5 elements of the scale being relevant to the cross-sectional studies. This approach was taken to allow an identical approach to quality assessment for prevalence estimates from cross sectional or (baseline of) longitudinal studies.”

  

- How was the variation in the reporting of adverse outcomes handled (for example, the differences in reporting functional decline across studies). Greater clarity is needed on the parameters used to extract data.

**Author response: **We have added the following text to the methods section to expand on this description along with a supplementary table of the extracted data which underlies the presentation of outcomes in the harvest plot: “For outcomes, we extracted data on the method used to assess the outcome, timeframe or length of follow-up, the magnitude of the association along with measure of uncertainty, and any adjustment for potential confounders. Where there was variation between studies in the assessment of similar outcomes we presented this data in supplementary tables.”

**Results**

- Overall, the findings to map the objectives of the review are clearly reported.

**Author response: **Thank you.

  

- It would be helpful to include more details in the narrative on the impact of the quality appraisal on the interpretation of findings.

**Author response: **Thank you for this suggestion. We have added the following text:

 “The studies assessing outcomes were judged to be representative of people with rheumatoid arthritis as most recruited consecutive or non-selected patients from rheumatology outpatient departments (where most patients with rheumatoid arthritis undergoing treatment are managed). Frailty measures were either validated or well-described. Cross sectional characteristics were assessed similarly in people with and without frailty. As such these were judged to be a high quality assessment of the cross-sectional associations between frailty and features of rheumatoid arthritis but with limited assessment of the longitudinal impact of frailty or the causal role of frailty in the development of outcomes and complications.”

**Discussion**

- Consideration needs to be given to other factors that might influence the prevalence of frailty aside from age and the measure of frailty?

**Author response: **We agree with the reviewer. We have added the middle sentence to the section below to emphasise the potential impact of additional factors on the prevalence of frailty: “This may reflect differences in the underlying population (e.g. ethnicity, socioeconomic status, disease activity), inclusion criteria, or the application of frailty measures. All of these factors may influence prevalence estimates of frailty. It is notable, therefore, that few studies reported data on ethnicity or socioeconomic status.”

  

- The results are well contextualised with regard to the current literature. The strengths and weaknesses are well-considered.

**Author response: **Thank you.

  

- Perhaps insert a few lines on the clinical implications of frailty in this population for a clinical audience.

**Author response: **Thank you. We have added the following text: “For clinicians, understanding that there is uncertainty around the prognostic significance of frailty in people with rheumatoid arthritis is important. Recommendations for frailty based, for example, on limited life expectancy or the likelihood of functional decline may not be relevant for all individuals with rheumatoid arthritis who meet the criteria for frailty. For this reason, future research assessing the relationship between frailty and outcomes such as mortality and the development of disability in people with rheumatoid arthritis, as disentangling this from the impact of rheumatoid arthritis disease activity, is important to inform clinical decisions.”

# Reviewer report: Reviewer response for version 1

**DOI:** 10.21956/wellcomeopenres.19016.r46327

**Published:** 2021-11-08

**Authors:** Dhammika Deepani Siriwardhana

**Affiliations:** 1 Research Department of Primary Care and Population Health, University College London, London, UK

This review summarizes the available evidence on the prevalence of frailty and associated adverse outcomes in people with rheumatoid arthritis. The manuscript is well written. I have a few comments for the authors to consider.

- In the abstract, the authors have mentioned that they searched in three electronic databases. However, in the methods, they have mentioned four databases (MEDLINE, EMBASE, WoS Core Collection, and Scopus). Suggest correcting the inconsistency.
- The objective (iv) (if frailty is associated with adverse health outcomes in the context of rheumatoid arthritis) is not clear. What was the rationale for this objective (potential clinical and research significance)?
- Authors could consider using the PRISMA 2020 diagram of study selection and checklist.
- Suggest mentioning for which database the search strategy presented in Box 1 was applied to.
- I wonder whether the authors have calculated the inter-rater reliability of the study selection/screening process.
- Was there any particular reason for allocating 2 points for the criteria “Ascertainment of exposure frailty” in the Newcastle-Ottawa tool?
- Are there any cut-offs to interpret the risk of bias assessment results?
- There are a few risks of bias assessment tools specifically developed for cross-sectional studies looking at several aspects of methodology and reporting results, e.g. AXIS, JBI critical appraisal tools for prevalence studies and analytical cross-sectional studies.
- What was the rationale for using a version of the Newcastle-Ottawa tool as a risk of bias assessment tool (only 5 criteria were applied to cross-sectional studies although the majority of the studies in this review are cross-sectional)?
- Suggest providing more information on data analysis, e.g. statistical software used to create figures, the information you fed to the software, etc.
- It is interesting that the authors have used harvest plots to present heterogeneous study findings. Was there any particular reason to annotate the bards of the harvest plots with sample size instead of using other characteristics, e.g. risk of bias assessment score?
- The method of identification of rheumatoid arthritis is not mentioned in the two included studies. I wonder whether the authors of this manuscript contacted the study authors for additional information.
- The following sentence is difficult to comprehend: “Most samples were recruited from rheumatology clinics, with most judged to be representative based on subsequent exclusion criteria and sampling methods.”.
- Table 1: “NA” stands for what? Suggest mentioning it as a footnote.
- Suggest incorporating minimum recruitment age for each study in Table 1.
- Suggest incorporating sample size into Figure 2.
- “Few studies presented data on non-responders.": Suggest presenting relevant references along with this statement.
- “Taken together these data show a consistent relationship between frailty and disease activity, assessed by a diverse range of measures.”: I wonder about the accuracy of the above claim since the findings are mixed at the moment.
- The authors have mentioned that they have provided the search strategy for different databases as a supplementary file in the PRISMA checklist. Unfortunately, I am not able to find it.
- “no data from upper-middle income of LMICs”: This phrase is not clear. Is it about “no data from upper-middle income countries of low-and middle-income countries” or “no data from low-and middle-income countries”?

Are the rationale for, and objectives of, the Systematic Review clearly stated?

Partly

Is the statistical analysis and its interpretation appropriate?

Yes

Are sufficient details of the methods and analysis provided to allow replication by others?

Partly

Are the conclusions drawn adequately supported by the results presented in the review?

Yes

Reviewer Expertise:

Public Health, Epidemiology

I confirm that I have read this submission and believe that I have an appropriate level of expertise to confirm that it is of an acceptable scientific standard, however I have significant reservations, as outlined above.

# Response

**Published:** 2022-08-05

**Authors:** Peter Hanlon

**Affiliations:** University of Glasgow, UK

Thank you for this review of our article. We have now uploaded a revised version based on these helpful comments and those of the second reviewer. Please see below for our response to each of the comments and details of changes added to the text. Thank you again for your time and consideration in reviewing our manuscript. 

 Review 1:

 This review summarizes the available evidence on the prevalence of frailty and associated adverse outcomes in people with rheumatoid arthritis. The manuscript is well written. I have a few comments for the authors to consider.

- In the abstract, the authors have mentioned that they searched in three electronic databases. However, in the methods, they have mentioned four databases (MEDLINE, EMBASE, WoS Core Collection, and Scopus). Suggest correcting the inconsistency.

**Author response:** Thank you. We have amended the abstract to correct this error.

  

- The objective (iv) (if frailty is associated with adverse health outcomes in the context of rheumatoid arthritis) is not clear. What was the rationale for this objective (potential clinical and research significance)?

**Author response: **We have changed the wording of this objective to make it clearer. It now reads: “what is the association between frailty and adverse health outcomes (e.g. hospitalisation or mortality) in people with rheumatoid arthritis.”

- Authors could consider using the PRISMA 2020 diagram of study selection and checklist.

**Author response: **We have updated the PRISMA diagram to the 2020 version.

  

- Suggest mentioning for which database the search strategy presented in Box 1 was applied to.

**Author response: **This was the search strategy used for Medline. We have added this to the title along with a note that the strategy was adapted for other databases.

  

- I wonder whether the authors have calculated the inter-rater reliability of the study selection/screening process.

**Author response: **We have now added this detail to the text: “Inter-rater agreement was high (kappa statistic 98%).”

  

- Was there any particular reason for allocating 2 points for the criteria “Ascertainment of exposure frailty” in the Newcastle-Ottawa tool?
- Are there any cut-offs to interpret the risk of bias assessment results?

**Author response:** We did not apply cut off nor was quality assessment used as an exclusion criteria. We have clarified this in the text. “Studies were not excluded on the basis of the quality assessment.”

  

- There are a few risks of bias assessment tools specifically developed for cross-sectional studies looking at several aspects of methodology and reporting results, e.g. AXIS, JBI critical appraisal tools for prevalence studies and analytical cross-sectional studies.

**Author response: **See the response to the comment below.

  

- What was the rationale for using a version of the Newcastle-Ottawa tool as a risk of bias assessment tool (only 5 criteria were applied to cross-sectional studies although the majority of the studies in this review are cross-sectional)?

**Author response: **This choice of the tool was made *a priori* before the identification of eligible studies. Our rationale was to allow a consistent application of quality assessment across all studies included in the review. This was particularly because among the aims of the review were to assess the prevalence and assess longitudinal outcomes. Where longitudinal studies were identified, we assessed prevalence using the baseline data from these studies. The Newcastle-Ottawa tool allowed a similar assessment to be made for cross-sectional studies and the baseline data of longitudinal studies (first 5 questions). While we acknowledge that no single quality assessment tool provides a complete or comprehensive assessment of study quality, this tool allowed us to explicitly show our judgments on representativeness, sample selection, frailty assessment, and outcome assessment. The quality of which is particularly central to the study aims and discussed in the accompanying text.

 We have added the following text to explain this decision:

 “The Newcastle-Ottawa scale is frequently used to assess quality of observational studies. Previous reviews have also adapted elements of the scale to reflect the studies of interest to the review itself. For this review, we used an adaptation previously developed for observational studies of frailty. This adaptation altered the ‘exposure’ component of the was altered to award two points if a study used validated measure of frailty implemented according to its original description. One point was awarded if studies used an alternative measure of frailty (e.g. an adapted or non-validated measure of frailty) but the criteria were described in sufficient detail to allow the assessment to be replicated. This adaptation was to reflect the fact that there is no ‘gold-standard’ measure of frailty and that frailty is assessed using a diverse range of measures within the literature. The scale was applied to all studies (cross sectional or longitudinal), with only the first 5 elements of the scale being relevant to the cross-sectional studies. This approach was taken to allow an identical approach to quality assessment for prevalence estimates from cross sectional or (baseline of) longitudinal studies.”

 And the following text to the limitation section: 

 “We used an adapted version of the Newcastle-Ottawa scale (prespecified in our protocol) to maximise the comparability of assessment of cross-sectional and longitudinal studies (e.g., where both assessed prevalence). However, most studies identified and included were cross sectional, and this tool is not specific to the assessment of cross-sectional studies.”

  

- Suggest providing more information on data analysis, e.g. statistical software used to create figures, the information you fed to the software, etc.

**Author response: **We have added the following sentences to the synthesis section of the methods: “Extracted data for each study were collected on .csv files.”

 “Prevalence estimates were plotted stratified by age-group of the sample and with reference to the frailty measure used for each estimate using the *ggplot2* package in R. Confidence intervals were calculated for each prevalence estimate using the point estimate and sample size.”

 “Harvest plots were generated using Microsoft Powerpoint.”

  

- It is interesting that the authors have used harvest plots to present heterogeneous study findings. Was there any particular reason to annotate the bards of the harvest plots with sample size instead of using other characteristics, e.g. risk of bias assessment score?

**Author response: **We intended the harvest plot to provide a visual summary of these data without cluttering the image with too much data. We have now added a supplementary table with the data underlying the harvest plot including the effect estimates themselves and the quality assessment scores for each study displayed.

  

- The method of identification of rheumatoid arthritis is not mentioned in the two included studies. I wonder whether the authors of this manuscript contacted the study authors for additional information.

**Author response:** We have gone back and evaluated these studies further. We were able to identify further detail on the respective cohorts from other publications which have allowed us to update Table 1 with this information.

  

- The following sentence is difficult to comprehend: “Most samples were recruited from rheumatology clinics, with most judged to be representative based on subsequent exclusion criteria and sampling methods.”.

**Author response:** Thank you for highlighting this. We have changed the sentence to the following to make it clearer: “We judged most of these to be representative of people with rheumatoid arthritis as most people with the condition will be managed within specialist outpatient clinics and the sampling techniques of these studies were generally inclusive without applying further, restrictive exclusion criteria.”

  

- Table 1: “NA” stands for what? Suggest mentioning it as a footnote.

**Author response: **These instances have now been removed from the table having clarified the method of RA definition in the two studies concerned.

  

- Suggest incorporating minimum recruitment age for each study in Table 1.

**Author response: **We have added this information.

  

- Suggest incorporating sample size into Figure 2.

**Author response: **Thank you, we have added the sample size to the study labels in this figure.

  

- “Few studies presented data on non-responders.": Suggest presenting relevant references along with this statement.

**Author response: **We have added references to this statement.

  

- “Taken together these data show a consistent relationship between frailty and disease activity, assessed by a diverse range of measures.”: I wonder about the accuracy of the above claim since the findings are mixed at the moment.

**Author response: **Thank you for highlighting this. We agree with the reviewer and have amended the sentence to the following: “Taken together these data show a consistent relationship between frailty and disease activity assessed using DAS28, however there was some inconsistency in this relationship when disease activity was assessed by different measures.”

  

- The authors have mentioned that they have provided the search strategy for different databases as a supplementary file in the PRISMA checklist. Unfortunately, I am not able to find it.

**Author response: **Thank you for highlighting. We have uploaded this with the revised version to the supplementary information available on the Zenodo link at the end of the manuscript.

  

- “no data from upper-middle income of LMICs”: This phrase is not clear. Is it about “no data from upper-middle income countries of low-and middle-income countries” or “no data from low-and middle-income countries”?

**Author response: **We have edited to “no data from low- and middle-income countries”
